# The “slide technique”—a novel free-hand method of subaxial cervical pedicle screw placement

**DOI:** 10.1186/s12891-020-03420-0

**Published:** 2020-06-23

**Authors:** Bin Liu, Xiangyang Liu, Xiongjie Shen, Guoping Wang, Yixin Chen

**Affiliations:** 1 Department of Spine Surgery, Hunan Provincial People’s Hospital (The First Affiliated Hospital of Hunan Normal University), Changsha, Hunan China; 2 Department of Rehabilitation, Xiangya Hospital of Central South University, Changsha, Hunan China

**Keywords:** Cervical spine trauma, Free-hand, Slide technique, Cervical pedicle screw (CPS)

## Abstract

**Background:**

Cervical Pedicle Screw (CPS) placement is a challenging work due to the high risk of neurovascular complications. Although there have been several different free-hand or navigation assisted techniques for CPS placement, perforations may occur during screw insertion, especially lateral perforation. The objective of this manuscript is to describe a novel free-hand technique for subaxial CPS placement (C3–C7) and to evaluate if it decreases the chances of perforation.

**Methods:**

Thirty-two patients undergoing surgery with CPS instrumentation (C3–C7) at our institute between June 2017 and December 2018 were included in this study. All the patients had cervical trauma, and pedicle screw insertion was performed according to the free-hand “slide technique”. The lamina, lateral mass and facet joint of the target area were exposed and the optimal entry point was found on the lateral mass posterior surface. A pedicular probe was then inserted and gently advanced. During the pedicle probe insertion, the cortex of the medial margin of the pedicle acted as a slide to permit the safe insertion of the screw. If the pedicle screw pathway was intact, the screw of the appropriate size was carefully placed. Three-dimensional (3D) CT imaging reconstruction was performed in all the patients after surgery, and screw perforations were graded with the Gertzbein-Robbins classification.

**Results:**

Thirty-two patients who met the inclusion criteria were included in this study. A total of 257 CPSs (C3–7) were inserted, of which 41 CPSs were in C3, 61 CPSs were in C4, 55 CPSs were in C5, 53 CPSs were in C6, and 47 CPSs were in C7. The diameter and length of CPSs were 3.5 mm and 22–26 mm respectively. According to the Gertzbein-Robbins classification, grade 0, 231 screws; grade 1, 19 screws; and grade 2, 7 screws. No neurovascular complications occurred stemming from malpositioning of pedicle screws. Among perforated screws (26 screws), there were 16 lateral perforations, 5 medical perforations, and 4 inferior perforations.

**Conclusions:**

The initial usage result shows the “slide technique” is a safe, effective and cost-effective technique for pedicle screw placement in the cervical spine. This is the first report of such a technique, and further studies are needed.

## Background

Due to the excellent three-column stability, pedicle screws have been widely used in spinal surgeries, including spinal fracture [1, 2], deformity [3], tumors [4], etc. Although pedicle screws have been routinely used for lumbar and thoracic fixation, the safety of its usage in the cervical spine remains a concern. Instead, spinous process wire, lateral mass screws or facet screws rather than cervical pedicle screws (CPSs) are commonly utilized during cervical posterior fixation.

It is a fact that anatomical structure and the adjacent relationship of the cervical spine are complex and dangerous, while the cervical spinal cord is close to the medial pedicle cortex, and the vertebral artery is close to the lateral pedicle cortex. CPS placement is a very challenging work due to the high risk of neurovascular complications, including critical bleeding, cerebral infarction, paralysis, and even death. Since Abumi et al. [5] first described the CPS placement method in 1994, many researchers have proposed various improved methods for this operation [6–10]. However, there is no ideal solution which can make CPS to be routinely used for cervical posterior fixation up to now.

With the advancement of technology in the field of digital navigation, the safety, as well as the accuracy of CPS placement, have been improved greatly [11]. However, the navigation system has its limitations, such as high cost [12], tedious procedures, and relatively limited indications [12].

Inspired by the “slide technique” in the thoracic spine [13], we devised a novel method for free-hand CPSs insertion also called “slide technique”, to increase the accuracy of CPS placement via a direct slide on the cortex of the medial margin of the pedicle. The purpose of this study is to describe the “slide technique” in CPS placement and to evaluate if it decreases the chances of perforation .

## Methods

### Study design

This was a clinical prospective study from June 2017 to December 2018. All the data were analyzed anonymously. An informed consent waiver was granted. This study was approved by the medical ethics committees of Hunan Provincial People’s Hospital (NO.2017-S65).

### Patients

Patients with cervical trauma underwent posterior cervical surgeries and the CPS instrumentation aimed at reconstructing cervical stability were included. Patients with cervical pedicle stenosis, dysplasia or severe destruction that cannot insert CPS were excluded.

A total of 32 patients who met the inclusion criteria were included in this study. All patients came from the department of spinal surgery, Hunan Provincial People’s Hospital. Their mean age was 52.8 years (range, 28–74 years). There were 23 male and 9 female patients.

### Surgical technique

All patients underwent a Three-dimensional (3D) Computed Tomography (CT) scan of the cervical spine to measure pedicle diameter and appropriate screw length before the operation.

**Fig. 1:**
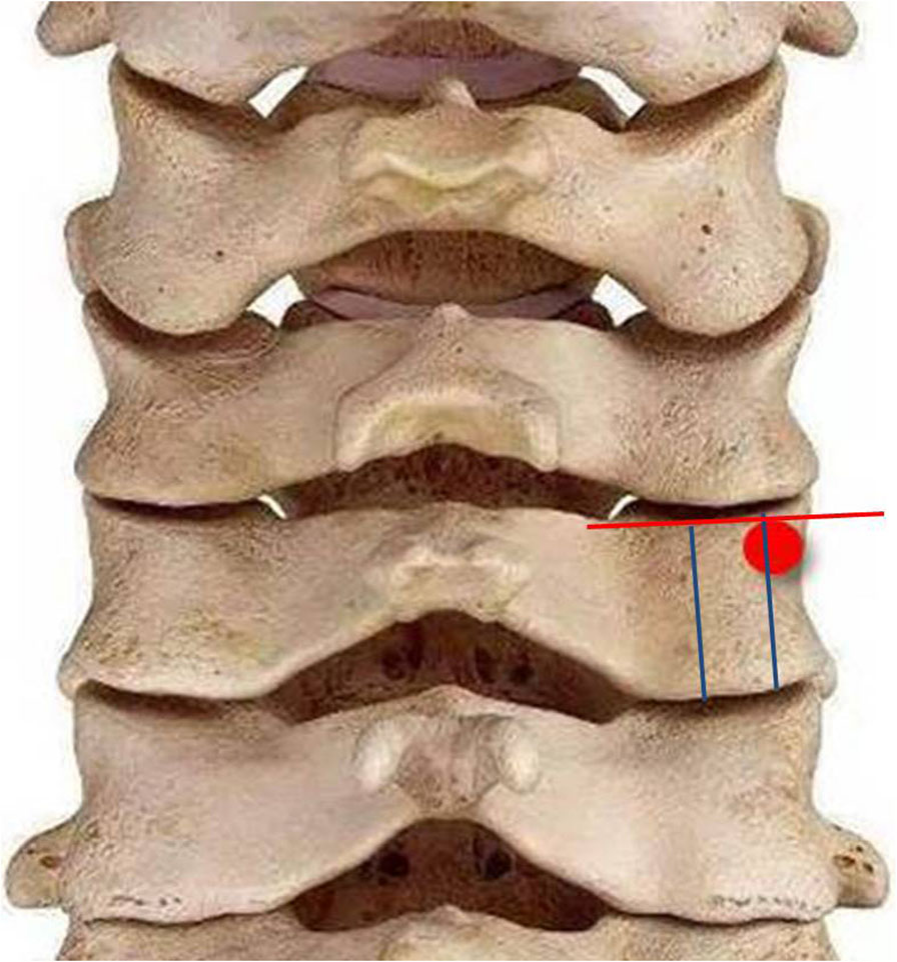
Optimal entry point (frontal view)

**Fig. 2:**
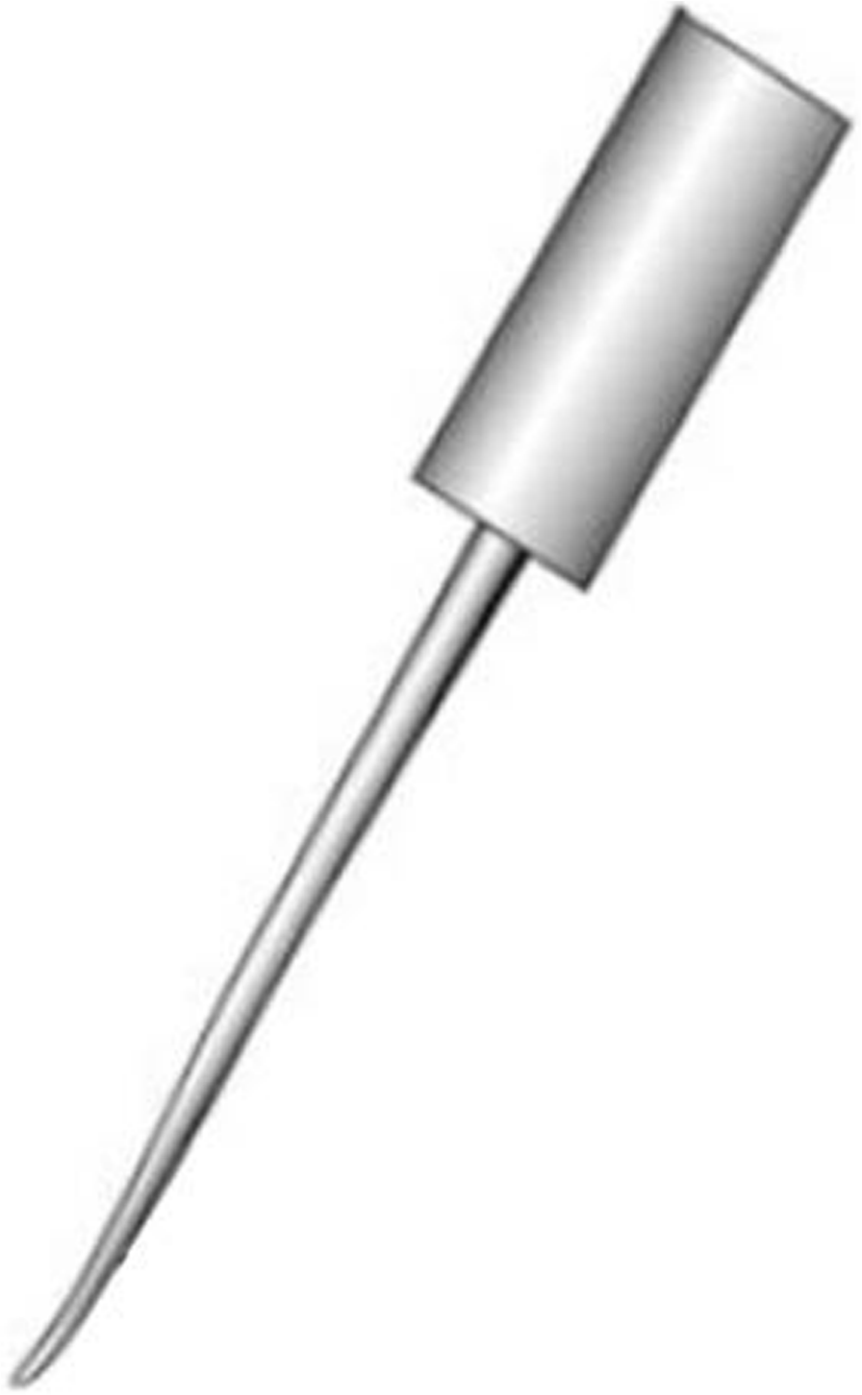
Diagram of the pedicle probe

**Fig. 3:**
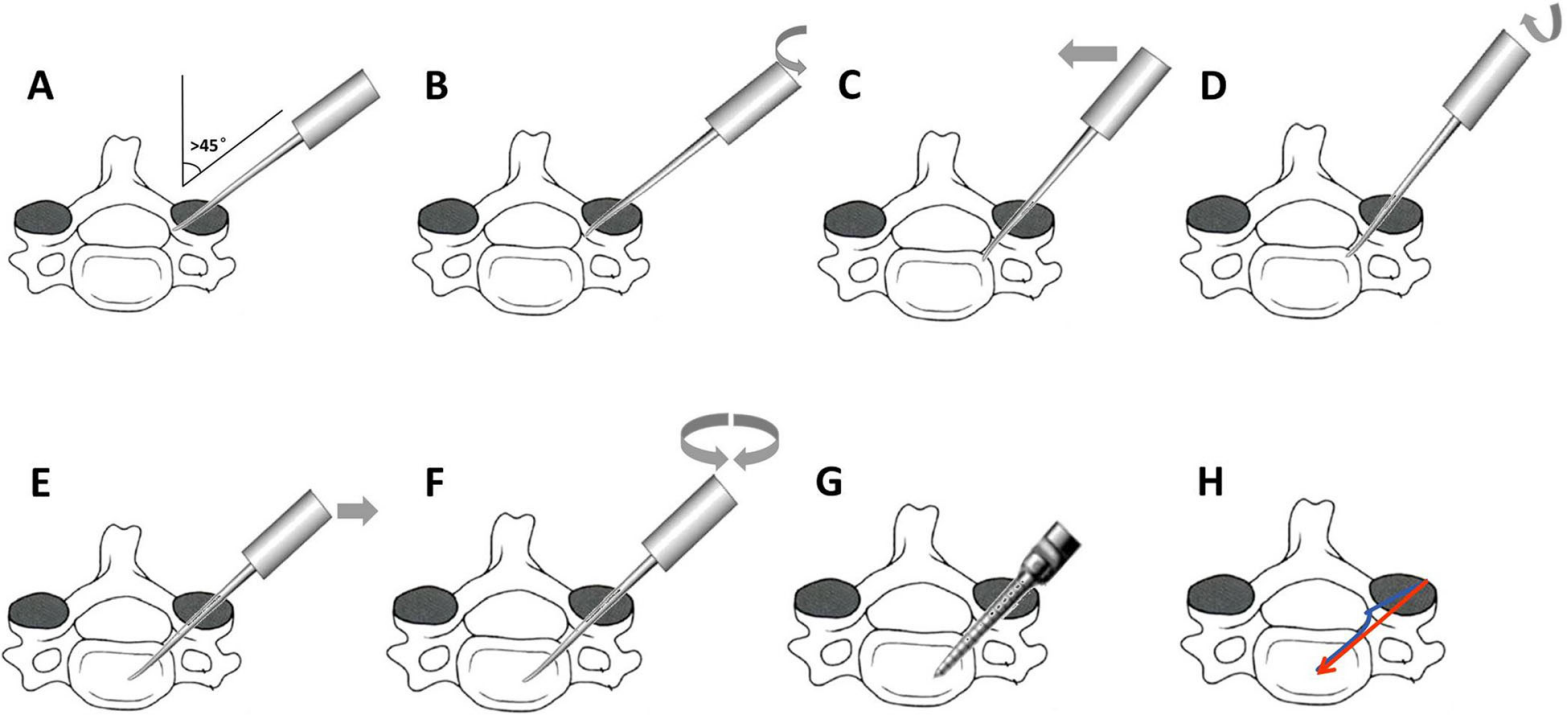
Operative technique (axial view)

All patients were placed in the prone position with the head fixed using brachiocephalic traction. After successful general anesthesia, we made a standard midline skin incision on the posterior neck. Subperiosteal dissection was performed along the spinous process and lamina. Lateral mass and facet joint of the target area were exposed. The target vertebral lateral mass was divided into three equal parts by longitudinal lines and the optimal entry point was chosen at a point on the lateral longitudinal line and slightly below the inferior margin of the facet joint. The distance between the inferior margin of the facet joint and entry point needed to be measured on X-ray or CT images before operation (Fig. 1). First, the cortex at the entry point was penetrated with a high-speed burr or awl. The pedicle probe was blunt-tipped with bent head, and its diameter was 2.1 mm(Fig. 2). After entered from the entry point with head bend inside, the pedicle probe rotated while gently advancing at a maximum angle on more than 45° medially. Probe stopped when meeting resistance. At this time, the probe head had reached the cortex of the medial margin of the pedicle (Fig. 3a). Thereafter, by rotating the probe by 180 (its head became to bend outward) (Fig. 3b) and reducing the medial angle, the pedicle probe avoided the medial cortex easily and it could continue to advance to the vertebrae body. During this procedure, the cortex of the medial margin of the pedicle acted as a slide to permit the safe insertion of the probe (Fig. 3c). Once the probe reached a depth of 15 mm (head of probe had reached the body), it was rotated by 180 ° again to make its head bend inside (Fig. 3d). After increasing the medial angle, the pedicle probe then continued to insert and gently advanced to a depth of 20 mm (Fig. 3e). We rotated the probe by 360° twice to expand the pathway (Fig. 3f). A ball-tip probe was used to evaluate the integrity of the pedicle screw pathway. If the pathway was intact, the screw of the appropriate size was carefully placed (Fig. 3g). The blue line in Fig. 3h represented the changes of the pathway during the probing, and the red line represented the final screw pathway. C-arm fluoroscopy was used to preliminarily evaluate the screw position during operation, especially to avoid superior and inferior perforations.

The key point of the “slide technique” was to use the cortex of the medial margin of the pedicle as a “slide” to permit correct probe positioning. The theoretical supports of this novel method includes 1. the cortex of the medial margin of the cervical pedicle was 1.4–3.6 times thicker than that of the lateral margin [14], so it was not easy to break through when negotiating, tapping and placing a screw. In comparison, the cortex of the lateral margin was thin and easy to be perforate. Previous studies had confirmed that the incidence rate of lateral perforation was significantly higher than that of the medial perforation during CPS insertion [15, 16]. 2. There were buffer tissues such as dural sac and epidural fat between the medial pedicle cortex and cervical spinal cord. Cases of medical perforation without spinal cord injury often could be seen in clinical work. In contrast, the vertebral artery closed to the lateral pedicle cortex was easy to be damaged due to the limitation of the vertebroarterial foramen. Therefore, we believed that it was safe and reliable to select the cortex of the medial margin of the pedicle for sliding.

### Evaluation of security and accuracy

3D-CT imaging reconstruction was performed in all the patients after cervical surgeries, to assess pedicle screw instrumentation. We observed whether CPSs penetrate the pedicle cortex or not and if so, measured the distance between screws and the pedicle cortex. According to Gertzbein-Robbins classification [17], the degree of perforation was classified into four grades on postoperative CT scans. If the CPS located within the pedicle and did not perforated, that was defined as grade 0. Grade 1 perforation was defined if perforation was less than 2 mm. If perforation was 2–4 mm, it was classified as grade 2. Perforation of more than 4 mm was defined as grade 3. We also recorded the direction of perforation, including lateral perforation, medical perforation, superior perforation, and inferior perforation.

Besides, we reviewed the clinical information of all patients. Complications directly related to CPSs placement were recorded in all patients, such as spinal cord injury, nerve root injury, vertebral artery injury/rupture, aortic injury, etc.

The sample size of our study was calculated from our clinical experience (α error, 0.05; power, 0.8; P 0.9; P_0_, 0.8). The required sample size was at least 108 CPSs. We used SPSS Version 22.0 statistical software for all analyses.

## Results

A total of 257 CPSs (C3–7) were inserted, of which 41 CPSs were in C3, 61 CPSs were in C4, 55 CPSs were in C5, 53 CPSs were in C6, and 47 CPSs were in C7. The diameter and length of CPSs were 3.5 mm and 22–26 mm respectively. According to the Gertzbein-Robbins classification, 231 screws (89.9%) were grade 0; 19 screws (7.4%) were grade 1; and 7 screws (2.7%) were grade 2. In perforated screws (26 screws), lateral perforations were 16(61.5%), medical perforations were 5(19.2%), and inferior perforations were 4(15.4%).

**Fig. 4:**
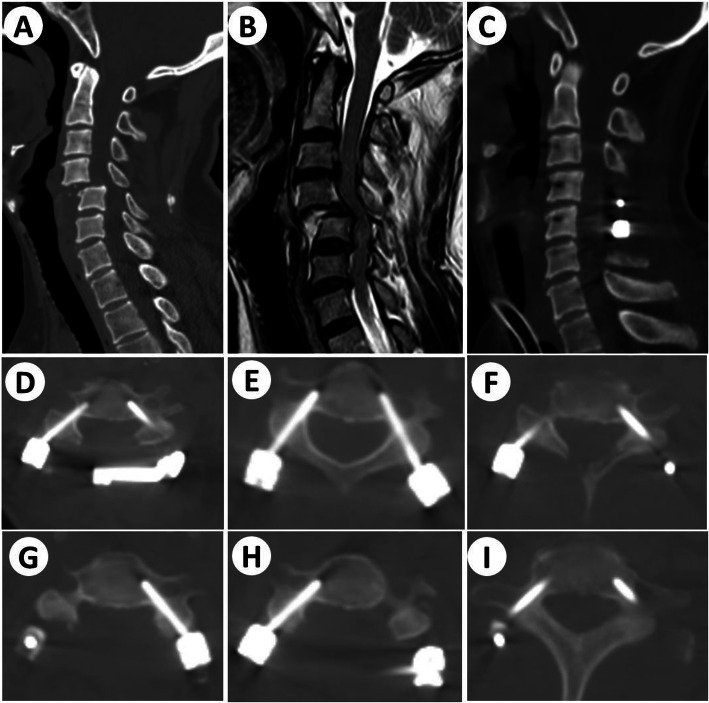
Example of a patient with cervical trauma. **a** Preoperative cervical CT scan, **b** Preoperative cervical MR imaging, **c** Sagittal CT image after cervical operation, **d-i** transverse CT images after cervical operation

No neurovascular complications occurred stemming from malpositioning of a pedicle screw during operation. None of these patients showed neurological deterioration after the surgery or symptoms related to vertebral artery injury/rupture. There was also no nerve root injury happened after CPSs placement. A typical case is shown in Fig. 4.

## Discussion

Cervical fixation using pedicle screws is becoming increasingly popular for various cervical diseases due to their advantages, such as three-column fixation, sagittal correction, and excellent biomechanical stability when compared with spinous process wire, lateral mass screws or facet screws [18, 19]. However, the CPS has not become the routine for posterior cervical fixation due to the high risk and high skills required.

In addition to the CPS, lateral mass screw (LMS) is another commonly used method for posterior subaxial cervical fixation. Previous researches and clinical practices showed that these two methods have their advantages and disadvantages [19]. The placement of LMS is comparatively simple, and the incidence of neurovascular complications during operation is lower [20]. However, LMS showed a small amount of bony purchase and thereby lesser pullout strength especially in the presence of osteoporosis in biomechanical experiments when compared with CPS. By contrast, the biomechanical result showed the pullout strength was significantly higher for the CPS, and thus it was relatively difficult to loosen [19]. The technique for CPS placement is more difficult than LMS although much successful clinical use of CPSs has been described. Reports of the CPS insertion failure have shown the possibility of serious iatrogenic injury [16, 21].

The most challenging process of placing CPSs safely into the cervical vertebrae is making the exact cancellous pathway between the vertebral artery and cervical spinal cord for the pedicle screw. Lateral pedicle perforation when placing CPSs may injury the vertebral artery, while medial pedicle perforations can injury the cervical spinal cord.

The cortex of the medial margin of the cervical pedicle is much thicker and stronger than that of the lateral margin, so it is predisposed to lateral perforation during pathway preparation, tapping, and insertion of the screws. Previous literature has confirmed this point of view. A multicenter study from Japan on the complications of CPS placing during the conventional free-hand technique showed that 75% (57/76) of all misplaced screws were lateral pedicle perforation, while only 25% (19/76) were medial pedicle perforation [16]. Therefore, attention needs to be paid to how to reduce lateral pedicle perforation while designing a new CPS placing technology.

The most representative conventional free-hand technique for subaxial CPS placing is the method proposed by Abumi et al. [5] in 1994. The contents include: the entry point at the posterior cortex of the articular mass was determined slightly lateral to the center of the articular mass and close to the posterior margin of the superior articular surface. The intended angle of the screws based on measurements of preoperative CT images was 30–40° medial to the midline in the transverse plane, and parallel to the upper end-plate in the sagittal plane. The insertion of the screw was greater than two-thirds of the AP vertebral body depth. Subsequently, Jeanneret [6], Miller [7], and Liu [8] proposed various improved free-hand methods for CPS placement. There were also studies reported the CPS placement using the medial funnel technique [22] and medial cortical pedicle screw technique [9]. Burcev et al. [10] introduced a standardized and fast method for subaxial CPS: screw insertion based on the simple angles to the bony landmarks. However, the accuracy of conventional CPS placement methods needs to be improved though there are many methods to choose from.

In recent years, digital navigation technology, 3D printing technology, and robotic technology are also arising in CPS placement. For example, Ishikawa et al. [23] placed 108 cervical CPSs using an intraoperative, full-rotation, 3D image (O-arm)-based navigation system. The results showed that 96 of them (88.9%) were grade 0, 9 were grade 1 and 3 were grade 2. There were no complications such as vascular and nervous complications, indicating that a combination of intraoperative 3D image-based navigation with other techniques may result in more accurate CPS placement. But not all the results about using the navigation in CPSs placement were optimistic. Uehara et al. [15] inserted the CPSs by using a CT-based navigation system during operations. The results showed that the combined rate of grades 2 and 3 perforations was 20.0% (116/579). Therefore, the authors concluded careful insertion of pedicle screws is necessary, especially at C3 to C5, even when using a CT-based navigation system. There were a few studies even showing more perforations with navigated screws than with the free-hand technique [24, 25]. Although most of the studies support that navigation technology can improve the accuracy of pedicle screw placement [11], none of the high-tech can completely avoid the occurrence of screw perforations. The navigation system and other computer-aided technology always associated with complex operation procedures and expensive equipment costs [12]. Moreover, due to the high mobility of the cervical spine, cervical spine alignment can easily change during operation [26], which may lead to inaccurate synchronization to preoperative images. All the shortcomings limit the application of digital navigation technology, especially in developing countries.

Therefore, it is of great clinical significance to develop a cost-effective and safe method for free-hand CPS placement. Raphael Vialle et al. [13] developed the “sliding technique” for safe pedicle screw placement in the thoracic spine in 2004. The key point of this novel technique was to use the cortex of the anterior aspect of the transverse process and the lateral margin of the pedicle as a “slide” to permit correct probe positioning during pedicle probe insertion. Inspired by it, we devised a novel method for free-hand CPSs insertion also called “slide technique”, to increase the accuracy of CPS placement via a direct slide on the cortex of the medial margin of the pedicle.

Preliminary clinical results of this novel technique showed a higher rate of correct screw position, comparable to CPS placement with navigation system. Meanwhile, no neurovascular complications occurred stemming from malpositioning of pedicle screws. In the process of this free-hand technique, screw perforations inevitably occurred, with lateral pedicle perforation accounted for the majority, which was consistent with previous studies.

Several points should be paid attention to in the clinical application of the “sliding technique”. First, it is necessary to carefully read and analyze the imaging data before an operation, especially to accurately measure the diameter of the pedicle and pay attention to the variation of anatomical structure (such as pedicle sclerosis, pedicle slenderness, vertebral foramen malformation, local bone destruction, etc.). Second, the pedicle probe should be rotated while gently being advanced, and be stopped in case of resistance. No violence should be used during the whole operation. To ensure the accuracy of the pathway, intraoperative fluoroscopy is necessary. Third, when CPS placement is difficult, lateral mass screw or other fixation may be selected.

Please note that the patients included in the current study were all with cervical trauma, whose pedicle variation rate was small, so the accuracy of screw placement was relatively easy to achieve. The accuracy of the screw may decrease when applied to difficult situations such as cervical deformity.

## Conclusions

To our knowledge, this is the first report of slide technique use in CPS placement. This novel free-hand “slide technique” could be considered as a safe, effective and cost-effective method for CPS placement.

However, this study is also limited due to the relatively small number of screws used and uniform criterion for pedicle perforation. Besides, all the CPSs placements were evaluated by the researcher involved in the surgery, which could have led to bias. A multicenter large sample study is desired to establish in the future.

## Data Availability

The datasets used and analysed in the current study are available from the corresponding author on reasonable request.

## Abbreviations

CPS: Cervical Pedicle Screw
3D: Three-Dimensional
CT: Computed Tomography
LMS: Lateral Mass Screw
